# Parent–Child Communication Incongruence in Pediatric Healthcare

**DOI:** 10.3390/children11010039

**Published:** 2023-12-28

**Authors:** Nancy Kwun Yiu Ng, Joanne Dudeney, Tiina Jaaniste

**Affiliations:** 1 Departments of Pain & Palliative Care, Sydney Children’s Hospital, Randwick, NSW 2031, Australia; kwun.ng@health.nsw.gov.au (N.K.Y.N.); joanne.dudeney@mq.edu.au (J.D.); 2 School of Clinical Medicine, University of New South Wales, Kensington, NSW 2033, Australia; 3 School of Psychological Sciences, Macquarie University, Macquarie Park, NSW 2109, Australia

**Keywords:** parent, pediatric, verbal communication, nonverbal communication, healthcare, incongruence

## Abstract

Parents play a key role in providing children with health-related information and emotional support. This communication occurs both in their homes and in pediatric healthcare environments, such as hospitals, outpatient clinics, and primary care offices. Often, this occurs within situations entailing heightened stress for both the parent and the child. There is considerable research within the communication literature regarding the nature of both verbal and nonverbal communication, along with the way in which these communication modalities are either similar (i.e., congruent) or dissimilar (i.e., incongruent) to one another. However, less is known about communication congruency/incongruency, specifically in parent–child relationships, or within healthcare environments. In this narrative review, we explore the concept of verbal and nonverbal communication incongruence, specifically within the context of parent–child communication in a pediatric healthcare setting. We present an overview of verbal and nonverbal communication and propose the Communication Incongruence Model to encapsulate how verbal and nonverbal communication streams are used and synthesized by parents and children. We discuss the nature and possible reasons for parental communication incongruence within pediatric settings, along with the consequences of incongruent communication. Finally, we suggest a number of hypotheses derived from the model that can be tested empirically and used to guide future research directions and influence potential clinical applications.

## 1. Introduction

Effective parent–child communication is complex, yet crucial for building healthy relationships between parents and their children. It plays a central role in a child’s emotional and behavioral development [1,2,3], exerting particular importance during times of stress [4,5,6]. Within pediatric healthcare environments (e.g., hospitals, outpatient clinics, primary care offices) the way in which parents communicate with their child about health-related content is known to have profound effects on a range of child psychological, behavioral, and medical outcomes [7,8,9,10,11,12,13,14]. Similarly, parent–child health-related communication is of importance in the home environment, potentially entailing information provision about medical conditions and past or forthcoming procedures, as well as communication aimed at supporting the child in his coping efforts. Doctors commonly communicate information directly to the parents, rather than to the child [15,16,17], with parents thereby serving as information brokers, requiring them to filter and share the information with their child in an age-appropriate way [15,18]. There is, therefore, a need to better understand the nature and challenges of parent–child health-related communication.

Broadly, parent–child communication comprises both verbal and nonverbal communication modalities. A comprehensive understanding of parent–child communication needs to take into account the interplay between the giving and receiving of both communication modalities [19]. The degree of similarity (i.e., congruence) or dissimilarity (i.e., incongruence) between verbal and nonverbal information is one factor proposed to impact on the effectiveness of the interaction [20,21], the perceived credibility of the communicator [22], and the clarity of the overall message [20]. The concept of verbal and nonverbal communication incongruence has been explored within the broader communication literature [20,23], but to date, it has not been applied in a pediatric healthcare context.

In the current narrative review, we outline the available literature on verbal and nonverbal communication incongruence and consider potential applications for parent–child health-related communication. A model which depicts the role of verbal and nonverbal communication between a parent and child is presented, and we apply this Communication Incongruence Model in the pediatric healthcare context. Next, we consider the health-related contexts in which parental incongruent communication may be more likely to occur and discuss the potential impacts of incongruent communication. We conclude with some future research directions to promote a deeper understanding of parent–child communication incongruence within pediatric healthcare contexts, along with potential clinical implications.

## 2. The Relationship between Verbal and Nonverbal Communication

Verbal communication is the use of spoken language to intentionally convey a message between two people, a sender and a receiver [19]. Nonverbal communication comprises acts of communication that do not involve spoken language, including facial expressions, gestures, body and eye movements, and paralinguistic speech, such as tone and timing [24,25,26]. It has been proposed that the relationship between verbal and nonverbal communication is based on a series of interactions between three streams of communication: (1) symbolic, (2) spontaneous, and (3) pseudo-spontaneous [19]. Symbolic communication involves the intentional use of symbols and is most commonly verbal in nature, such as describing a forthcoming medical procedure. A shared knowledge of language must exist between two people for this interaction to be successful, such as speaking the same language, or using language that is developmentally-appropriate for a child. Spontaneous communication, however, involves the *involuntary* display of nonverbal signs, reflecting underlying thoughts and emotions (e.g., frowning as a sign of concern). The third stream of communication, pseudo-spontaneous, is also nonverbal and occurs when nonverbal signs are *intentionally manipulated* to demonstrate certain thoughts and emotions. For example, parents may intentionally manipulate their facial expressions and posture to try to appear calm prior to their child’s surgery, even though they feel quite anxious.

**Figure 1:**
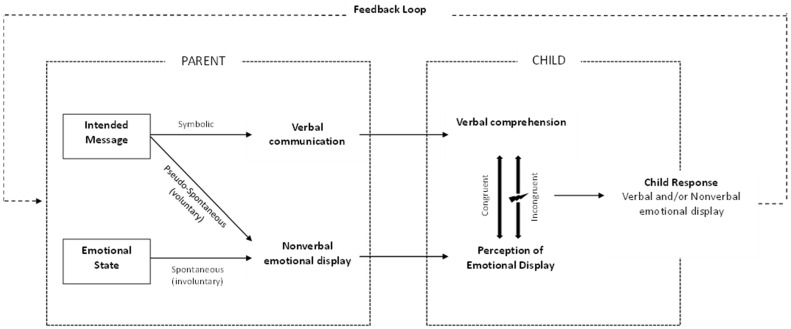
Communication Incongruence Model depicting communication between a parent and child.

Buck and VanLear [19] proposed a model outlining the communication process between two people, a sender and receiver, entailing symbolic, spontaneous and pseudo-spontaneous communication. In the current review, we have adapted the model by encapsulating the way in which a child synthesizes the streams of communication from his/her parent, depending on whether the communication is congruent or incongruent, while also highlighting the bidirectional flow of parent–child communication (see Figure 1).

As the model indicates, a parent communicates to his/her child via verbal information and nonverbal emotional displays (e.g., facial expression, body stance, and posture). Verbal information conveys the parent’s intended message to the child (e.g., “there is nothing to worry about”, “the doctors need to do another biopsy”). Nonverbal emotional displays usually convey a direct spontaneous display of the parent’s emotional state, which may or may not match their verbal communication. For example, parents intending to reassure a child may say that there is “nothing to worry about”, yet their spontaneous facial expressions reflect the parents’ concern. This example illustrates verbal and nonverbal incongruence. It is proposed, however, that parents may have some influence over their nonverbal emotional display, such as putting on a positive or neutral expression while verbally reassuring their child, in spite of the parents’ own anxieties. This is an example of a pseudo-spontaneous display, meaning that the nonverbal emotional display is manipulated to match the intended verbal message, and this is an example of verbal and nonverbal congruence.

From the child’s perspective, what has been communicated by the parent, both verbally and nonverbally, is usually interpreted as an overall message [27]. Thus, when verbal and nonverbal communication modalities are congruent, the overall message is coherent and more easily interpreted [20,28]. However, when there is verbal and nonverbal incongruence, there is room for confusion, misinterpretation, or the need for a forced choice between which content to process. It has been suggested that in cases of incongruence, the receiver may give one channel of information primacy, processing that content preferentially [23,29,30,31]. Given the decoding and analysis skills required for understanding verbal information, some researchers have proposed that primacy is often given to nonverbal channels [19,31]. Others, however, have suggested [32] or shown [23] that the relative importance, and thus primacy, of information may be context specific. Specifically, verbal information may be more important for factual, task-, or achievement-based contexts or in persuasive communication, whereas nonverbal displays may be more important for relational or affective messages which convey an attitude or emotion [23].

Regardless of whether the information communicated is congruent or incongruent, children interpret (or misinterpret) their parents’ verbal and nonverbal communication and respond through their own verbal communication and/or nonverbal emotional display. As depicted in the model, we propose that the child’s response serves as a feedback loop, influencing the ongoing nature of the parent’s message. For example, parents may tailor their next message accordingly, if they observe that the child is showing signs of, or uses language consistent with, negative emotions.

When considering the communication processes between a parent and child, as depicted in the Communication Incongruence Model, developmental factors must also be considered. This includes the child’s ability to decode or comprehend verbal communication, interpret nonverbal displays, and integrate and make sense of the combined message. Language comprehension in children is dependent on a combination of knowledge and skills [33]. Language development in typically developing children begins in infancy and is the greatest between 2 and 6 years of age [34]. However, health-specific language may be largely dependent on experience.

The ability to interpret facial expressions is thought to emerge within the first year of a child’s life [35,36,37,38], and it continues to develop throughout childhood until the age of about 14 years [39]. It is well documented in the literature that happiness is accurately recognized early on (preschool age and younger), with recognition of more complex emotions, such as fear and disgust, taking longer to mature [40,41,42,43,44]. Research has also shown that children as young as four possess the ability to recognize emotions from non-verbal displays extending beyond facial expressions (e.g., body expression through movement), with decoding ability improving with age [45]. Studies have further found that young children can differentiate conflicting verbal–nonverbal information, such as tone of voice and facial expression [46,47], to form their own understanding of the overall message.

## 3. Parental Verbal–Nonverbal Incongruence within the Pediatric Healthcare Setting

Parents are commonly required to make decisions about what health-related information to share with their child. In situations of chronic or serious medical conditions, parents also have to consider how they communicate with well-siblings of the sick child, who turn to their parents for information [48]. It is not uncommon for parents to minimize or withhold verbal information that is distressing, either to themselves or to their child [18]. Parents may also strive to self-regulate and/or mask their own negative emotions, such as by putting on a brave face in the hospital waiting room. Arguably, however, in situations of elevated stress, parents may lack the necessary resources to convey pseudo-spontaneous, nonverbal signs that match their verbal communication. When attempting to conceal high intensity emotions, there is a greater likelihood of unintended signs of the underlying emotions showing (i.e., spontaneous nonverbal displays) [49]. Thus, parents may make verbal comments intended to reassure the child, but nonverbal signs, such as worried expressions or fidgeting, may lead to incongruent communication. This incongruent communication may seem confusing and can be difficult for the child reconcile [20]. Given that many pediatric healthcare contexts may be highly stressful for the parent, incongruent communication may be more likely in these situations than in other environments where parent–child communication occurs.

In Table 1, we hypothesize the conditions or reasons why parental verbal–nonverbal incongruence may occur within the pediatric healthcare setting. Broadly, communication incongruence may arise in situations in which parents intentionally manipulate their verbal message, but give less attention to their nonverbal communication. Conversely, incongruence may also occur if parents are more conscious of their nonverbal displays, not wanting to display their genuine emotions, and perhaps not want to burden or worry their child. In such cases, parents may focus on manipulating their nonverbal signs, but if they are highly distressed, they may be less able to plan their verbal communication. Table 1 describes particular situations in which incongruent communication may result.

## 4. Potential Effects of Incongruent Communication

There is a paucity of research investigating the effects of incongruent communication in youth, and to our knowledge, no research investigating the effects of parental incongruent communication. In regards to the small number of studies in youth, the findings suggest that the effects are largely negative. Incongruent communication has been associated with reduced attentional engagement, diminished credibility of the speaker, and reduced believability/trustworthiness [22,50,51,52,53]. For example, Gillis and Nilsen [22] found children aged 7 to 8 rated congruent speakers as more believable than incongruent speakers. In contrast, children aged 4 to 5 only rated the congruent speakers more believable than the incongruent speakers if the congruent speakers communicated positively, highlighting a developmental progression in the impact of communication incongruence. Moreover, children aged 7 to 8 demonstrated a preference to solicit further information from a somewhat familiar adult who used congruent communication, rather than from a speaker who used incongruent communication or from an unknown/unfamiliar adult [22]. Similarly, another study found that children aged 9 to 10 were able to take into account situational context when determining whether incongruent messages could be relied on, whereas 7- to 8-year-olds did not demonstrate such flexibility [54]. Rotenberg, Simourd, and Moore [53] also examined the extent to which 5-, 7- and 9-year-olds perceived an adult (non-parental) speaker as truthful, based on whether they perceived the verbal and nonverbal communication as congruent. The results showed a developmental progression in the extent to which children used the verbal–nonverbal congruency principle to identify whether the speaker was telling the truth. Five-year-olds rarely used the verbal–nonverbal congruency of the speaker to detect truth from lying, whereas 9-year-olds consistently used the congruency of the information to draw conclusions about the truthfulness of the adult [53].

If the results of the above studies were to generalize to the pediatric healthcare context, one may speculate that children, especially older children, may be more likely to believe their parent and/or to ask further questions, if the information (e.g., a new diagnosis, preparation for a procedure) is communicated congruently. Conversely, incongruent health-related communication may result in children being less engaged in the conversation, which parents may interpret as a lack of interest or readiness to receive the information, and parents may consequently be less likely to share further information. However, further research in this area is required to understand how communication incongruence affects the parent–child relationship within pediatric healthcare, and also to evaluated the developmental trajectory of how children synthesize and understand verbal–nonverbal communication congruence.

## 5. Future Research and Clinical Directions

The proposed Communication Incongruence Model is derived from concepts in the broader communication literature, offering numerous testable hypotheses pertinent to the pediatric healthcare environment. Parents are required to communicate health-related information and provide support to their children in a variety of contexts (e.g., before medical procedures or surgery, when receiving a diagnosis, and at the beginning of a new treatment). Future research should assess the type of healthcare situations in which parental verbal and nonverbal communication modalities are congruent and to examine a range of parent- and child-factors that may be associated with the level of (in-)congruence, including contextual factors (e.g., fatigue, stress), individual difference factors (e.g., emotional self-regulation, catastrophizing), and cultural factors (e.g., emotional expression, nonverbal displays). The specific impacts of parental verbal–nonverbal incongruent communication within the context of health-related communication are yet to be explored. Research is needed to explore the possible associations between parental incongruence in the healthcare context and subsequent child anxiety and the adherence to health behaviors, as well as in the context of the understanding and memory of health-related information. With emerging literature exploring parent–child health-related narratives [55,56] and their impact on children’s memory of these events [57], communication (in-)congruence within parental narratives provides a possible mechanism which may impact children’s memory of health-related events and information. Specifically, if verbal and nonverbal communication is incongruent in parental narratives, one may hypothesize that the overall message may not be encoded as effectively by the child, resulting in poorer subsequent recall. This suggestion remains to be studied.

The implications of parental verbal–nonverbal communication incongruence for the longer-term parent–child relationship also needs to be examined. For example, the perceived trustworthiness of the parent, the likelihood for the child to elicit further health-related information from the parent via the feedback loop proposed in the model, reciprocal child incongruent communication, and its impact on the overall parent–child relationship all warrant consideration through longitudinal studies.

Developmental considerations regarding the impact of parental communication incongruence should be explored. Evidence from stress-free, experimental settings suggests that 7- and 8-year-olds have greater awareness of when speakers are displaying incongruent communication, relative to 4- and 5-year-olds [22]. Older children are also more likely to infer a lack of credibility in incongruent speakers [53], but have a greater capacity to consider the situational context when evaluating speaker incongruence [54]. However, similar studies examining age-effects have not been conducted with parent–child dyads, nor in situations of potentially heightened stress, such as pediatric healthcare environments. Furthermore, the role of parent and child gender has not been investigated in terms of possible associations with incongruent communication and its impacts.

If the negative impacts of verbal and nonverbal incongruence are found to generalize to the pediatric healthcare context, strategies designed to minimize parental verbal and nonverbal incongruence would be valuable. Such strategies would need to be related to the many, varied reasons why parents engage in incongruent communication with their children. For example, interventions may potentially need to target poor parental emotional self-regulation, limited parental awareness of their nonverbal displays, parental anxiety management, or parental strategies to respond to the child’s distress response. It is possible that screening in such areas may help to identify which parents are most likely to engage in incongruent communication. However, further research is needed before it can be confirmed that this is a fruitful avenue to pursue.

Psychoeducation and skills training (e.g., role-plays, watching videos of congruent/incongruent parent–child interactions, mindfulness-based strategies) may be potentially effective strategies to increase parental knowledge of, and awareness regarding, any incongruence in their communication. Since acceptance- and mindfulness-based interventions are designed to promote an awareness and acceptance of one’s current internal experience [58,59], such interventions may lend themselves to fostering congruent communication by heightening the parents’ awareness and acceptance of their emotional experiences and nonverbal displays. However, the use of these clinical interventions for such purposes requires further research before they can be recommended for these intentions.

Finally, research is required to determine whether incongruent verbal–nonverbal communication may be an underlying mechanism for why parental reassurance statements (e.g., “*It will all be okay*” or “*You will be fine*”) have been widely documented to be unhelpful for children in the context of medical procedures, resulting in higher pain and distress outcomes [7,8,60]. If the parents’ reassuring verbal statements are at odds with their own thoughts or emotions (e.g., worry that the procedure is likely to be highly painful), there may be a spontaneous, unintended display of negative emotions, resulting in verbal and nonverbal communication incongruence. This may, in turn, affect how the child interprets the information and situation, resulting in negative outcomes. However, this remains to be tested.

### Research Paradigms

In considering the broad range of future research goals outlined above, it would be useful to consider research paradigms that are amenable for this type of research in the pediatric healthcare context. Naturalistic, observational research paradigms have the ability to take advantage of the unique stressors that are present in a healthcare context to directly examine their effects on parent–child communication [11,14]. For example, video-recordings of parent–child interactions in hospital or medical contexts may permit the detailed coding of verbal and nonverbal emotional displays, allowing researchers to determine the level of directional and reciprocal incongruence in health contexts. Observational coding scales, such as the Iowa Family Interaction Rating Scale [61] or the Child–Adult Medical Procedure Interaction Scale-Revised [62], could be adapted to include codes for verbal and nonverbal congruency/incongruency [63]. The above scales were developed to assess and categorize verbal and nonverbal parent–child interactions and have been used with pediatric populations [10,62,64,65].

A paradigm that has been used in other fields to understand parent–child interactions involves parents watching recordings of their interactions with their children and verbalizing the thoughts and feelings they remember arising throughout the interactions [66,67]. Such a research paradigm would help researchers to understand the parents’ motivations behind their verbal messages and nonverbal emotional displays, enabling empirical exploration of the possible reasons for parental incongruence, outlined in Table 1, identifying whether parents were consciously manipulating either channel of communication.

Another approach could be to place parent–child dyads from healthy and illness-related populations in a manipulated situation to observe and code their interactions to simulated health-related or other stressors. Versions of these tasks have been used previously in studies of parent–child incongruence research [68,69], as well as in research into child attachment [70,71,72], anxiety [73,74], and asthma [75]. Within an experimentally manipulated situation, researchers could empirically investigate communication incongruence between parents and children who are unwell, versus children who are healthy, to compare how parents communicate when a stressor is health-related versus non-medical. This will allow us to begin to understand the role of the health-context, and health-stressors, in how parent–child interactions play out in regards to verbal and nonverbal communication incongruency.

## 6. Conclusions

Verbal and nonverbal communication incongruence is an important aspect of parent–child communication, and it may have particular relevance in the pediatric healthcare context. Parents are more prone to engage in incongruent communication in situations of elevated stress, such as current or impending pediatric medical procedures. Parents may minimize or withhold verbal information, aiming to minimize the child’s stress (or their own), but they may lack the necessary resources to adjust their nonverbal communication accordingly. As outlined in this paper, the resulting incongruent communication is likely to be confusing for the child, with potential implications regarding the credibility of the parent. Based on the Communication Incongruence Model, we have outlined a research agenda with a particular focus on parent–child communication in the healthcare setting. Further research may yield clearer clinical applications.

## Figures and Tables

**Table 1:** Potential reasons for parental incongruent verbal and emotional communication.

Manipulating the intended verbal message	
(a) Belief that it is not in the child’s best interests to share full, accurate information.	The parent believes the child is not capable of understanding the information. The parent believes the child is not capable of managing the information emotionally (e.g., opting not to tell a child that his/her sibling’s cancer is terminal). The parent wants to minimize the child’s distress (e.g., avoiding telling the child about a forthcoming surgery or poor prognosis). The parent believes that the information does not concern the child (e.g., not telling the child much about his/her sibling’s illness).
(b) The preference is to avoid information-giving.	The parents believe themselves incapable of managing their own emotions (e.g., a parent who has just learned that his/her child has a poor prognosis may avoid sharing this information with their kids if the parent worries that her/she will be too upset to explain things to the children). The parents believe themselves incapable of managing their child’s emotional response.
(c) The focus is predominantly on verbal content	The parents may practice what they will say (especially with a serious topic, such as a forthcoming medical procedure or a serious diagnosis) and may have less awareness of their nonverbal, emotional displays.
2. Manipulating the emotional display	
(a) The parents are motivated to self-regulate their own emotions.	The parent wants to avoid “spilling” their own negative emotions onto the child. The parents may lack the coping skills to manage their own negative emotions effectively and therefore, may attempt to conceal or mask their negative emotions (i.e., emotion suppression). The parents may want to appear a certain way, so they manipulate their self-image (e.g., parents may try to appear calm and in control, even if they are upset about some worrying medical information). The parents are not accepting of their own emotions and thus try to conceal them.
(b) If parents are pre-occupied with managing their own negative emotions, they may be less able to focus on their verbal communication.	If the parents are highly distressed, they may be less able to regulate their verbal communication in a way that is best suited to the needs of the child; parental verbal communication may be impulsive and poorly planned.
(c) The parents may enhance their emotional display to gain support.	The parents may seek to elicit certain responses from their child, such as gaining the child’s assistance with tasks, or leaving them alone, e.g., a child may be less likely to bother the parents with other requests if the parents convey that they are feeling overwhelmed or upset).

## References

[1] Vaccari C., Marschark M. (1997). Communication between parents and deaf children: Implications for social-emotional development. J. Child Psychol. Psychiatry.

[2] Runcan P.L., Constantineanu C., Ielics B., Popa D. (2012). The role of communication in the parent-child interaction. Procedia Soc. Behav. Sci..

[3] Ackard D.M., Neumark-Sztainer D., Story M., Perry C. (2006). Parent-child connectedness and behavioral and emotional health among adolescents. Am. J. Prev. Med..

[4] Alderfer M.A., Long K.A., Lown E.A., Marsland A.L., Ostrowski N.L., Hock J.M., Ewing L.J. (2010). Psychosocial adjustment of siblings of children with cancer: A systematic review. Psycho-Oncology.

[5] Cohodes E.M., McCauley S., Gee D.G. (2021). Parental buffering of stress in the time of COVID-19: Family-level factors may moderate the association between pandemic-related stress and youth symptomatology. Res. Child Adolesc. Psychopathol..

[6] Tang S., Xiang M., Cheung T., Xiang Y.-T. (2021). Mental health and its correlates among children and adolescents during COVID-19 school closure: The importance of parent-child discussion. J. Affect. Disord..

[7] McMurtry C.M., Chambers C.T., McGrath P.J., Asp E. (2010). When “don’t worry” communicates fear: Children’s perceptions of parental reassurance and distraction during a painful medical procedure. Pain.

[8] McMurtry C.M., McGrath P.J., Chambers C.T. (2006). Reassurance can hurt: Parental behavior and painful medical procedures. J. Pediatr..

[9] Rodriguez E.M., Dunn M.J., Zuckerman T., Hughart L., Vannatta K., Gerhardt C.A., Saylor M., Schuele C.M., Compas B.E. (2013). Mother–child communication and maternal depressive symptoms in families of children with cancer: Integrating macro and micro levels of analysis. J. Pediatr. Psychol..

[10] Murphy L.K., Rodriguez E.M., Schwartz L., Bemis H., Desjardins L., Gerhardt C.A., Vannatta K., Saylor M., Compas B.E. (2016). Longitudinal associations among maternal communication and adolescent posttraumatic stress symptoms after cancer diagnosis. Psycho-Oncology.

[11] Chorney J.M., Tan E.T., Kain Z.N. (2013). Adult–child interactions in the postanesthesia care unit. Anesthesiology.

[12] Chorney J.M., Torrey C., Blount R., McLaren C.E., Chen W.-P., Kain Z.N. (2009). Healthcare provider and parent behavior and children’s coping and distress at anesthesia Induction. Anesthesiology.

[13] Blount R.L., Corbin S.M., Sturges J.W., Wolfe V.V., Prater J.M., Denise James L. (1989). The relationship between adults’ behavior and child coping and distress during BMA/LP procedures: A sequential analysis. Behav. Ther..

[14] Spagrud L.J., Von Baeyer C.L., Ali K., Mpofu C., Fennell L.P., Friesen K., Mitchell J. (2008). Pain, distress, and adult-child interaction during venipuncture in pediatric oncology: An examination of three types of venous access. J. Pain Symptom Manag..

[15] Lee S.P., Haycock-Stuart E., Atan A., Shamsuddin N.A. (2023). Understanding Parental Role in Children’s Participation in Decision Making during Hospitalisation: An Ethnographic Study in Malaysia. Malays. J. Med. Sci..

[16] Tates K., Meeuwesen L. (2001). Doctor–parent–child communication. A (re)view of the literature. Soc. Sci. Med..

[17] van Dulmen A.M. (1998). Children’s contributions to pediatric outpatient encounters. Pediatrics.

[18] Young B., Dixon-Woods M., Windridge K.C., Heney D. (2003). Managing communication with young people who have a potentially life threatening chronic illness: Qualitative study of patients and parents. BMJ.

[19] Buck R., VanLear C. (2002). Verbal and nonverbal communication: Distinguishing symbolic, spontaneous, and pseudo-spontaneous nonverbal behavior. J. Commun..

[20] Grebelsky-Lichtman T. (2014). Parental patterns of cooperation in parent-child interactions: The relationship between nonverbal and verbal communication. Hum. Commun. Res..

[21] Green J.O., Whaley B.B., Samter W. (2007). Formulating and Producing Verbal and Nonverbal Messages: An Action Assembly Theory. Explaining Communication: Contemporary Theories and Exemplars.

[22] Gillis R.L., Nilsen E.S. (2017). Consistency between verbal and non-verbal affective cues: A clue to speaker credibility. Cogn. Emot..

[23] Grebelsky-Lichtman T. (2017). Verbal versus nonverbal primacy: Children’s response to parental incongruent communication. J. Soc. Pers. Relatsh..

[24] Mandal F.B. (2014). Nonverbal communication in humans. J. Hum. Behav. Soc. Environ..

[25] Duncan S. (1969). Nonverbal communication. Psychol. Bull..

[26] Hall J.A., Horgan T.G., Murphy N.A. (2019). Nonverbal communication. Annu. Rev. Psychol..

[27] Jones S.E., Lebaron C.D. (2002). Research on the relationship between verbal and nonverbal communication: Emerging integrations. J. Commun..

[28] Ekman P., Friesen W.V. (1974). Detecting deception from the body or face. J. Pers. Soc. Psychol..

[29] Friedman H.S. (1978). The relative strength of verbal versus nonverbal cues. Pers. Soc. Psychol. Bull..

[30] Bugental D.E., Kaswan J.W., Love L.R. (1970). Perception of contradictory meanings conveyed by verbal and nonverbal channels. J. Pers. Soc. Psychol..

[31] Mehrabian A., Wiener M. (1967). Decoding of inconsistent communications. J. Pers. Soc. Psychol..

[32] Burgoon J.K., Guerrero L.K., Floyd K. (2016). Nonverbal Communication.

[33] Hagen Å.M., Melby-Lervåg M., Lervåg A. (2017). Improving language comprehension in preschool children with language difficulties: A cluster randomized trial. J. Child Psychol. Psychiatry.

[34] Melby-Lervåg M., Lervåg A., Lyster S.-A.H., Klem M., Hagtvet B., Hulme C. (2012). Nonword-repetition ability does not appear to be a causal influence on children’s vocabulary development. Psychol. Sci..

[35] Bayet L., Pascalis O., Gentaz É. (2014). The development of emotional facial expression discrimination by infants in the first year of life. Année Psychol..

[36] Grossmann T. (2010). The development of emotion perception in face and voice during infancy. Restor. Neurol. Neurosci..

[37] Palama A., Malsert J., Gentaz E. (2018). Are 6-month-old human infants able to transfer emotional information (happy or angry) from voices to faces? An eye-tracking study. PLoS ONE.

[38] Nelson C.A. (1987). The recognition of facial expressions in the first two years of life: Mechanisms of development. Child Dev..

[39] Kolb B., Wilson B., Taylor L. (1992). Developmental changes in the recognition and comprehension of facial expression: Implications for frontal lobe function. Brain Cogn..

[40] Boyatzis C.J., Chazan E., Ting C.Z. (1993). Preschool children’s decoding of facial emotions. J. Genet. Psychol..

[41] Camras L.A., Allison K. (1985). Children’s understanding of emotional facial expressions and verbal labels. J. Nonverbal Behav..

[42] Widen S.C., Russell J.A., Lewis M., Haviland-Jones J.M., Barrett L.F. (2008). Young Children’s Understanding of Others’ Emotions. Handbook of Emotions.

[43] Widen S.C. (2013). Children’s interpretation of facial expressions: The long path from valence-based to specific discrete categories. Emot. Rev..

[44] Widen S.C., Russell J.A. (2013). Children’s recognition of disgust in others. Psychol. Bull..

[45] Boone R.T., Cunningham J.G. (1998). Children’s decoding of emotion in expressive body movement: The development of cue attunement. Dev. Psychol..

[46] Lyons-Ruth K. (1977). Bimodal perception in infancy: Response to auditory-visual incongruity. Child Dev..

[47] Volkmar F.R., Siegel A.E. (1979). Young children’s responses to discrepant social communications. J. Child Psychol. Psychiatry.

[48] Jaaniste T., Tan S.C., Aouad P., Trethewie S. (2020). Communication between parents and well-siblings in the context of living with a child with a life-threatening or life-limiting condition. J. Paediatr. Child Health.

[49] Porter S., Brinke L., Wallace B. (2012). Secrets and lies: Involuntary leakage in deceptive facial expressions as a function of emotional intensity. J. Nonverbal Behav..

[50] Morioka S., Osumi M., Shiotani M., Nobusako S., Maeoka H., Okada Y., Hiyamizu M., Matsuo A. (2016). Incongruence between verbal and non-verbal information enhances the late positive potential. PLoS ONE.

[51] Van Buren A. (2002). The relationship of verbal-nonverbal incongruence to communication mismatches in married couples. N. Am. J. Psychol..

[52] Bugental D.E., Lyon J.E., Lin E.K., McGrath E., Bimbela A. (1999). Children “tune out” in response to the ambiguous communication style of powerless adults. Child Dev..

[53] Rotenberg K.J., Simourd L., Moore D. (1989). Children’s use of a verbal-nonverbal consistency principle to infer truth and lying. Child Dev..

[54] Gillis R.L., Nilsen E.S., Gevaux N.S. (2019). Children accept information from incongruent speakers when the context explains the communicative incongruence. Cogn. Dev..

[55] Pavlova M., Mueri K., Peterson C., Graham S., Noel M. (2022). Mother- and father-child reminiscing about past events involving pain, fear, and sadness: Observational cohort study. J. Pediatr. Psychol..

[56] Pavlova M., Orr S.L., Noel M. (2020). Parent-child reminiscing about past pain as a preparatory technique in the context of children’s pain: A narrative review and call for future research. Children.

[57] Noel M., Pavlova M., Lund T., Jordan A., Chorney J., Rasic N., Brookes J., Hoy M., Yunker W.K., Graham S. (2019). The role of narrative in the development of children’s pain memories: Influences of father- and mother-child reminiscing on children’s recall of pain. Pain.

[58] Waltz T.J., Hayes S.C., Kazantzis N., Reinecke M.A., Freeman A. (2010). Acceptance and Commitment Therapy. Cognitive and Behavioral Theories in Clinical Practice.

[59] Grossman P., Niemann L., Schmidt S., Walach H. (2004). Mindfulness-based stress reduction and health benefits: A meta-analysis. J. Psychosom. Res..

[60] Manimala M.R., Blount R.L., Cohen L.L. (2000). The effects of parental reassurance versus distraction on child distress and coping during immunizations. Child. Health Care..

[61] Melby J.N., Conger R.D., Kerig P., Lindahl K. (2001). The Iowa Family Interaction Rating Scales: Instrument Summary. Family Observational Coding Systems: Resources for Systematic Research.

[62] Blount R.L., Cohen L.L., Frank N.C., Bachanas P.J., Smith A.J., Manimala M.R., Pate J.T. (1997). The Child-Adult Medical Procedure Interaction Scale–Revised: An assessment of validity. J. Pediatr. Psychol..

[63] Chorney J.M., McMurtry C.M., Chambers C.T., Bakeman R. (2015). Developing and modifying behavioral coding schemes in pediatric psychology: A practical guide. J. Pediatr. Psychol..

[64] Brown E.A., De Young A., Kimble R., Kenardy J. (2019). Development and validity of the Burns-Child Adult Medical Procedure Interaction Scale (B-CAMPIS) for young children. Burns.

[65] Dunn M.J., Rodriguez E.M., Miller K.S., Gerhardt C.A., Vannatta K., Saylor M., Scheule C.M., Compas B.E. (2011). Direct observation of mother–child communication in pediatric cancer: Assessment of verbal and non-verbal behavior and emotion. J. Pediatr. Psychol..

[66] Lawrence P.J., Davies B., Ramchandani P.G. (2013). Using video feedback to improve early father-infant interaction: A pilot study. Clin. Child Psychol. Psychiatry.

[67] Beebe B. (2003). Brief mother–infant treatment: Psychoanalytically informed video feedback. Infant Ment. Health J..

[68] Grebelsky-Lichtman T. (2014). Children’s verbal and nonverbal congruent and incongruent communication during parent-child interactions. Hum. Commun. Res..

[69] Grebelsky-Lichtman T., Shenker E. (2019). Patterns of nonverbal parental communication: A social and situational contexts approach. J. Soc. Pers. Relatsh..

[70] Shiller V.M., Izard C.E., Hembree E.A. (1986). Patterns of emotion expression during separation in the strange-situation procedure. Dev. Psychol..

[71] Koren-Karie N., Oppenheim D., Dolev S., Yirmiya N. (2009). Mothers of securely attached children with autism spectrum disorder are more sensitive than mothers of insecurely attached children. J. Child Psychol. Psychiatry Allied Discip..

[72] Ainsworth M.D.S., Bell S.M. (1970). Attachment, Exploration, and Separation: Illustrated by the Behavior of One-Year-Olds in a Strange Situation. Child Dev..

[73] Gar N.S., Hudson J.L. (2008). An examination of the interactions between mothers and children with anxiety disorders. Behav. Res. Ther..

[74] Hudson J.L., Rapee R.M. (2001). Parent—Child interactions and anxiety disorders: An observational study. Behav. Res. Ther..

[75] Sicouri G., Sharpe L., Hudson J.L., Dudeney J., Jaffe A., Selvadurai H., Hunt C. (2017). Parent-child interactions in children with asthma and anxiety. Behav. Res. Ther..

